# Hypoxic marker CA IX and adhesion mediator β-catenin are downregulated by lymphocytic choriomeningitis virus persistent infection

**DOI:** 10.18632/oncotarget.24387

**Published:** 2018-02-02

**Authors:** Andrea Fabianova, Monika Barathova, Lucia Csaderova, Veronika Simko, Miriam Zatovicova, Martina Labudova, Jaromir Pastorek

**Affiliations:** ^1^ Institute of Virology, Biomedical Research Center, Slovak Academy of Sciences, Bratislava 845 05, Slovak Republic; ^2^ Department of Chemistry, Faculty of Natural Sciences, University of SS. Cyril and Methodius, Trnava 917 01, Slovak Republic

**Keywords:** carbonic anhydrase IX, lymphocytic choriomeningitis virus, renal cell carcinoma, internalization, immunotherapy

## Abstract

Renal cell carcinoma is one of the most frequent cancer diseases with high resistance to radio- and chemotherapy. Mutation of VHL gene is frequent in these tumors leading to simulation of hypoxic conditions. Lymphocytic choriomeningitis virus, belonging to RNA viruses, is a neglected human pathogen and teratogen. We have found that infection of renal cell carcinoma cells by lymphocytic choriomeningitis virus strain MX causes a decrease of carbonic anhydrase IX protein and RNA level. Lower expression of carbonic anhydrase IX on the cell surface provides less target for carbonic anhydrase IX-targeted immunotherapy. What more, reduced levels of adhesion mediating protein β-catenin as well as E-cadherin, as a consequence of infection, suggest a possible increase in metastatic potential of cells infected by lymphocytic choriomeningitis virus strain MX. These results might help elucidate differences in patients susceptibility to immunotherapy directed against carbonic anhydrase IX or in developing new therapeutical strategies. Our data indicate that presence of infection can significantly affect patient response to cancer therapy.

## INTRODUCTION

Lymphocytic choriomeningitis virus (LCMV) is a RNA virus from Arenaviridae family. Virus is comprised of two RNA segments encoding four proteins. Viral nucleoprotein (NP) is the main protein which encapsidates the viral RNA and is the most expressed protein in infected cells. Glycoprotein precursor (GPC) is postranslationally cleaved into two glycoproteins (GP1 and GP2) [1, 2]. Z protein (ZP) containing a RING finger domain is a structural protein with some regulatory functions [3]. RNA-dependent RNA polymerase (L), together with NP and RNA, forms a minimal replication unit, the ribonucleoprotein (RNP) [1, 3].

Persistent infection caused by LCMV is characterized by high production of viral NP, low expression of GPs and absence of complete infectious virion production. Also LCMV-specific defective interfering particles are formed [4]. The completion of the virion is not possible because GP is not present on the cell membrane. Thus, virus is not able to spread by typical virus-receptor dependent way and instead is transmitted by cell-to-cell contacts utilizing keratin 1 [5]. Spreading by cell-to-cell contacts is more advantageous and effective than the classical transmission.

LCMV causes a persistent infection in common house mice (Mus musculus) and also pet rodents (hamsters, guinea pigs). Humans are infected by inhalation of aerosols from rodent excretes [6–8]. The prevalence of LCMV has an extensive geographical range, and the virus infects large numbers of humans. In the United States and Europe, the prevalence of LCMV in wild mice ranges between 3–20% [9] and in human sera between 1–9.1% [9–14]. However, in our previous study, we have recorded a 37.5% prevalence of LCMV antibodies in human sera in Bratislava, Slovakia [15]. Similar results were recorded in Croatia, where the prevalence was 36% [16].

The infection in humans is asymptomatic, or it might be presented with a whole palette of symptoms, from flu-like symptoms to severe encephalitis. The main concern is the asymptomatic presentation of LCMV infection in donors of organs for transplantation. Using organs from infected donors may have fatal consequences [17–19]. During donor organ transport, when hypoxia occurs, the virus may reactivate from persistence and cause fatal infection in immunosuppressed recipients [20]. Hypoxia is a recognized stimulus for LCMV reactivation, when infectious virions are released from the cells as is typical for acute or productive chronic infection [20]. The mechanism by which this LCMV reactivation is regulated is still not known, however the possibility of regulation by hypoxia inducible factor 1 (HIF-1) has not been ruled out [20].

HIF-1 is a transcription factor that plays the main role in cellular adaptation to lack of oxygen. Under normoxic conditions, key proline residues of its α subunit (HIF-1α) are hydroxylated by a family of oxygen-dependent hydroxylases [21], and HIF-1α undergoes ubiquitin-mediated degradation [22, 23]. Von Hippel-Lindau tumor suppressor gene (VHL) is a component of the E3 ubiquitin ligase complex implicated in the ubiquitination and degradation of α subunit of HIF-1α [22, 24, 25]. In hypoxia unhydroxylated HIF-1α is not capable of binding VHL and accumulates in the cell [26, 27].

Kidney cancer is among most frequently occurring cancers in western communities. It is diagnosed in more than 330,000 people each year worldwide, and accounts for over 140,000 deaths annually [28]. Approximately 90% of kidney cancers are renal cell carcinomas (RCCs) that develop in the renal parenchyma [29], with conventional clear cell RCC (ccRCC) being the most common (70–80%) histological type [30]. Somatic mutations or epigenetic alternations of VHL are observed in >80% of ccRCC [31, 32]. A modest proportion (2–4%) of RCC is associated with VHL syndrome caused by germline mutations in VHL [33]. All renal tumors bearing VHL mutations have a defective ubiquitination of HIF-1α [22, 23]. This leads to oxygen level-independent stabilization of HIF-1α. Thus, transcription factor HIF-1 is permanently active in VHL-mutated cells. HIF-1 is responsible for hypoxia-dependent regulation of a number of genes associated with angiogenesis, vascular reactivity and remodeling, glucose and energy metabolism, cell proliferation and survival, erythropoiesis and iron homeostasis [34]. One of the many genes regulated by HIF-1 is carbonic anhydrase IX (CA IX) [35].

CA IX is a transmembrane, enzymatically active metalloprotein expressed in majority of tumor tissues, but absent from normal tissues [36–42]. Carcinomas of the cervix, kidneys, colon, breast or lungs are tumors with highest expression of CA IX. CA IX is present in more than 80% of primary and metastatic RCCs, in 95 to 100% of ccRCCs, and absent or minimally expressed in normal tissues [43, 44]. Presence of CA IX in the tumor is a sign of bad prognosis, mainly because of its resistance to conventional radio- or chemotherapy [41, 45]. However, on the other hand, decreased CA IX levels and progression of kidney cancer indicates that reduced CA IX expression in this tumor type may be linked with better treatment outcome [79].

Despite many approved treatment regimens improving patients survival, treatment resistance still occurs, and metastatic RCC remains incurable. Recently, targeting CA IX by G250 mediated immunotherapy has shown promising results. Clinical trial in non-metastatic ccRCC patients treated with chimeric monoclonal G250 antibody Rencarex showed prolonged disease-free survival of about 22 months in patients with high CA IX score [46]. In Phase I and II, Rencarex (www.wilex.de) showed good safety and tolerability and promising efficacy. Ongoing Phase III study (ARISER) aims to assess the effect of adjuvant treatment on overall disease-free survival in RCC patients with high risk of recurrence of the disease after nephrectomy (www.wilex.de). Also, the chimeric G250 monoclonal antibody has the highest reported uptake in solid renal tumors. However, the fact that intratumoral distribution of the antibody is highly heterogenous, as observed also in other tumors, might limit the efficacy of immunotherapy [47]. G250 can induce receptor-mediated internalization [48] with prolonged intracellular persistence. The antibody-antigen interaction remains undisturbed and the complex can be recycled back to the cell surface in its intact form with preserved Fc part of the G250 antibody [49]. Also, recycling and exposure of G250 with intact Fc fragment on cell surface can prolong ADCC response, which represents its principal anticancer mode of action. Recycling of intact G250 also explains its long-lasting effect in patients [50].

A set of monoclonal antibodies specific to CA IX was also developed in our laboratory [51]. One of the promising antibodies with the same conformational epitope as G250 is the VII/20 antibody. Monoclonal antibody VII/20, which recognizes a conformational epitope on catalytic domain of CA IX and retains internalization ability, has promising properties for immunotherapy [52]. Approximately 30% of bound antibody is internalized in 3 h, and the level of internalized antibody is proportional to the amount of antibody attached to the cell surface [52].

In our study, we have described the influence of LCMV on ccRCC cells, which exhibit a permanent pseudohypoxic state due to the VHL mutation. We observe the effect of LCMV infection on the expression of HIF-1α and HIF targets, as well as on different properties of tumor cells. Among others, LCMV infection significantly affects the amount of CA IX protein. This finding may have implications for treatment of RCC patients, as the amount of CA IX on the cell membrane might influence susceptibility to therapy with anti-CA IX antibodies.

## RESULTS

### RCC4 cell line can be persistently infected with LCMV strain MX

Previous studies showed that hypoxia contributes to reactivation of LCMV strain MX. VHL-mutated RCC4 cells simulate conditions of hypoxia. We therefore wanted to investigate the impact of persistent LCMV infection on the expression of certain hypoxia-regulated proteins.

VHL mutation is characteristic for renal clear cell carcinoma. Our experiments were performed with VHL-deficient renal carcinoma cell line RCC4 and RCC4 VHL line with reintroduced wild-type VHL [53]. Under normoxic conditions, HIF-1α is hydroxylated and wild-type VHL marks HIF-1α for proteasomal degradation. In cells with VHL-deficiency, HIF-1α cannot be marked for degradation and accumulates inside cytosol also in normoxia. Stabilization of HIF-1α thus leads to the formation of a functional HIF-1 transcription factor under normal oxygen levels.

**Figure 1:**
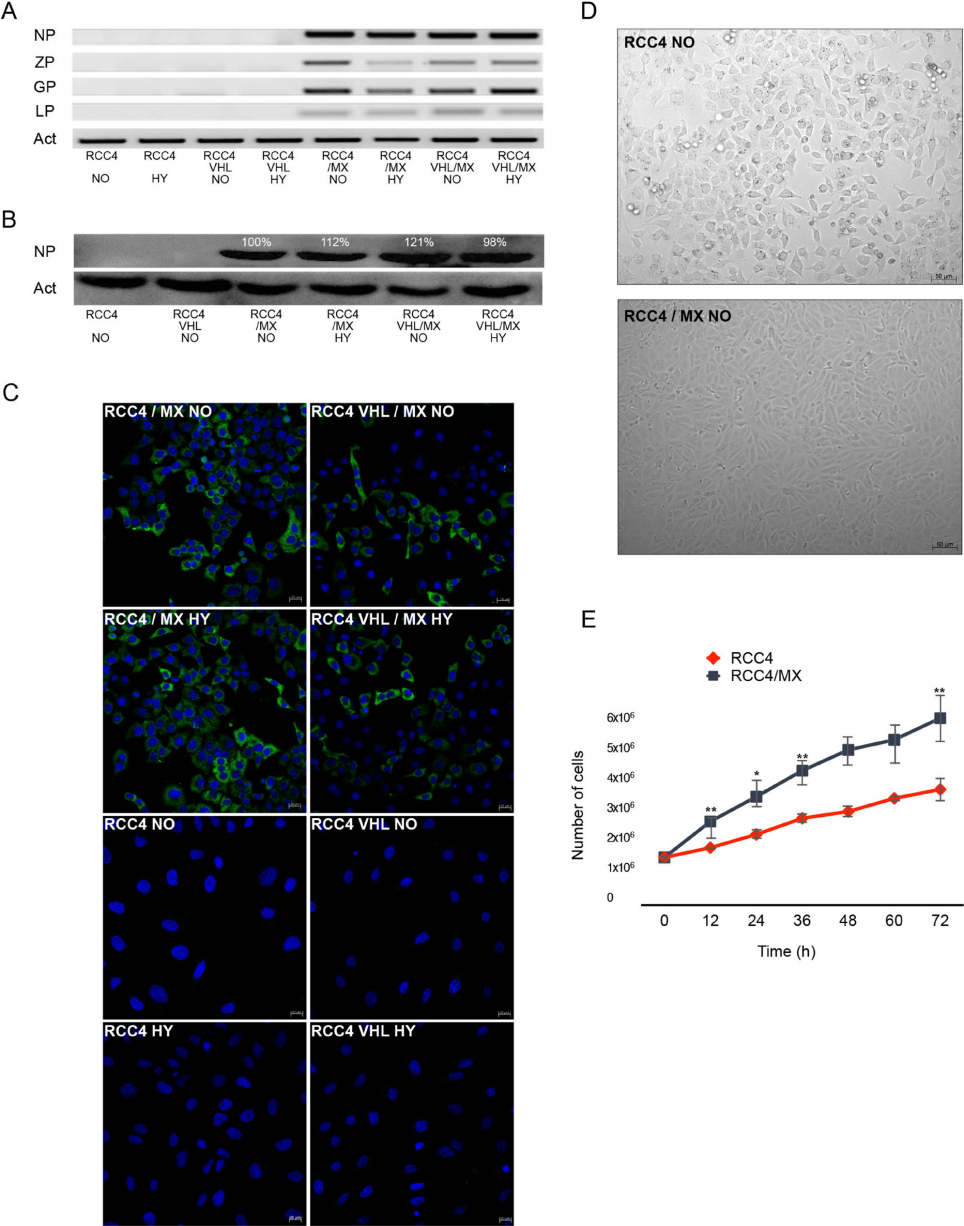
LCMV MX strain infection of RCC4 and RCC4 VHL cell lines (**A**) PCR of all viral genes, nucleoprotein (NP), Z protein (ZP), glycoprotein (GP), and L protein (LP), present in uninfected or infected RCC4 and RCC4 VHL cells (RCC4, RCC4 VHL, RCC4/MX, RCC4 VHL/MX) cultivated in normoxic (NO) or hypoxic (HY) conditions. (**B**) Western blot analysis for presence of viral NP in uninfected and infected RCC4 and RCC4 VHL cells cultivated in normoxic (NO) or hypoxic (HY) conditions. β-actin (Act) was used as a loading control. The numbers indicate percentage of densitometric evaluation. (**C**) Immunofluorescence of NP in infected or uninfected cells. Bar = 20 μm. (**D**) Transmitted light microscopy of uninfected RCC4 and infected RCC4/MX cells shows the changes of morphology caused by infection. Images were acquired by Microscope Zeiss Axiovert 40 CFL, magnification 200×. Bar = 50 μm. (**E**) Proliferation assay of RCC4 and RCC4/MX. Cells (103 cells/well) were plated into 24 well plate and counted every 12 hours. Y-axis shows the number of cells determined by cell counter at the indicated time points (X-axis).

RCC4 and RCC4 VHL cell lines were infected by cell-free extracts from BHK/MX cells [54]. We have used the RCC4 VHL cell line as a control to verify whether the ability of infection by LCMV is independent of the VHL status. The infection was verified by PCR for the presence of viral genes (Figure 1A) and also by western blot (Figure 1B) and immunofluorescence (Figure 1C) for the presence of NP. The presence of NP could be detected already in the second passage by both western blot and immunofluorescence. We also proved the presence of other viral genes, ZP, L and GP in both infected cell lines by PCR (Figure 1A). As L protein is the least abundant protein in infected cells, its presence was detected only in later stages of infection, while in the early passages it was missing. With these results, we have thus proven that the RCC4 and RCC4 VHL cell lines could be infected by the MX strain of LCMV, and this infection is not affected by the VHL mutation.

Moreover, after the infection, morphology of both cell lines changed (Figure 1D) as did their proliferation rates (Figure 1E). The morphology of infected cells changed from typical fibroblast-like to epithelial-like cells and accelerated their proliferation. The doubling time for the uninfected cells was approximately 17 h, while for the infected cells only 14 h.

### LCMV infection affects the expression of HIF-1 targets in the RCC4 cell line

VHL mutation present in the RCC4 cell line leads to activation and stabilization of HIF-1α subunit in normoxic conditions. HIF-1 induces expression of hypoxia-regulated genes including *CA9*. Therefore, we decided to study the impact of LCMV/MX on the expression of CA IX, HIF-1α and other selected HIF-1 targets in infected and uninfected RCC4 cells, under normoxic and hypoxic conditions.

**Figure 2:**
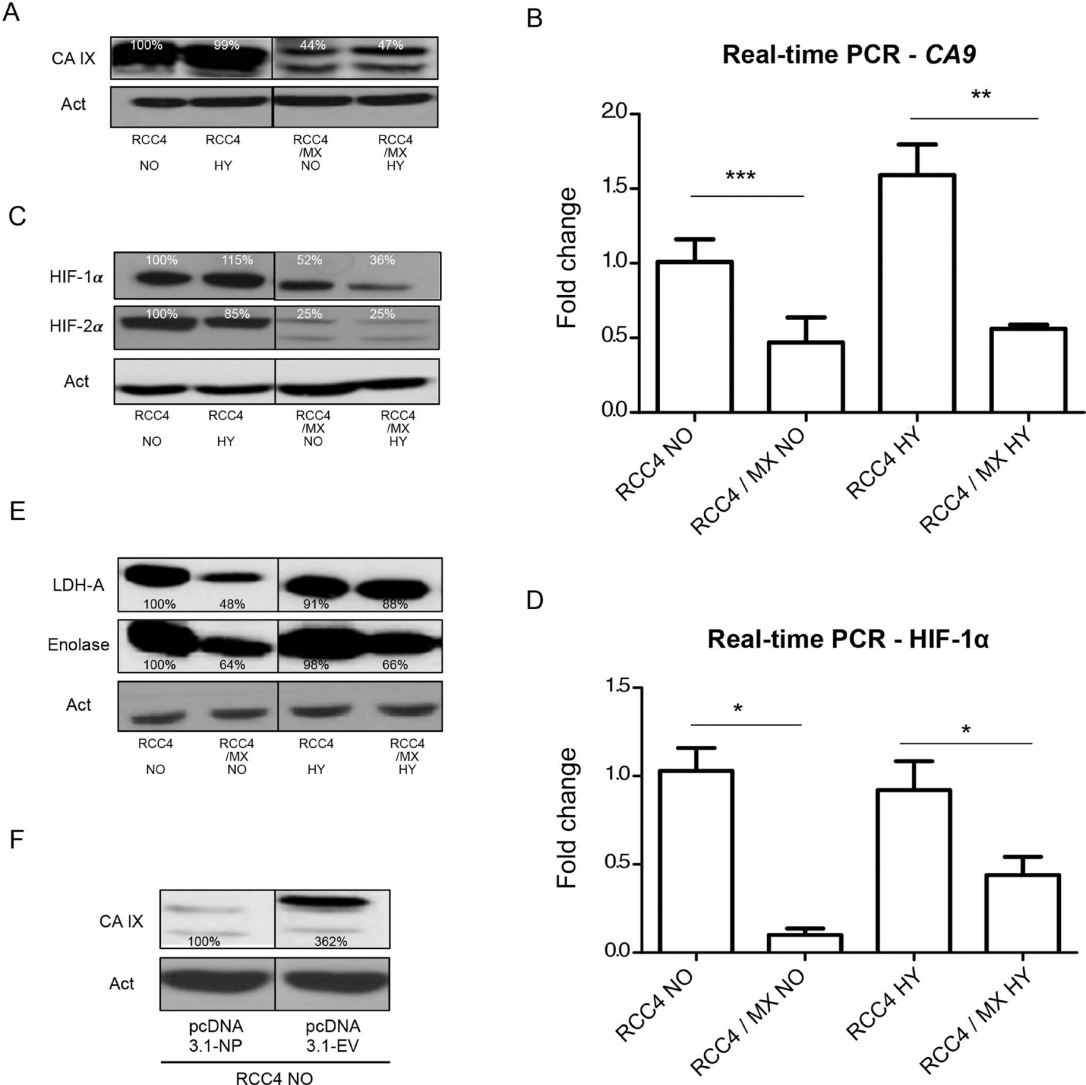
Changes in the expression of HIF-1 targets after infection with LCMV MX or transfection with pcDNA3.1-NP Western blot analysis was used to evaluate the levels of (**A**) CA IX, (**C**) HIF-1 α and HIF-2α and (**E**) HIF-1 targets, lactate dehydrogenase-A (LDH-A) and enolase (ENO) in uninfected RCC4 and infected RCC4/MX cells cultivated under normoxic (NO) and hypoxic (HY) conditions. β-actin (Act) was used as a loading control. The numbers indicate percentage of densitometric evaluation. To quantify the mRNA levels of (**B**) CA IX or (**D**) HIF-1 α in infected or control cells in normoxic (NO) and hypoxic (HY) conditions, a q-PCR with gene specific primers was done. The samples were normalized to β-actin and ΔΔCt method was used to evaluate the fold change in infected and uninfected cells. Single, double, and triple asterisk indicates *p* value lower than 0.05, 0.01, and 0.001, respectively, determined by the Student's *t*-test. (**F**) Western blot analysis of CA IX after transfection. Uninfected RCC4 cells were transfected by pcDNA3.1 empty vector (pcDNA3.1-EV) and pcDNA3.1-NP plasmids and subjected to western blot analysis. β-actin (Act) was used as a loading control. The numbers indicate percentage of densitometric evaluation.

We confirmed very strong expression of the CA IX protein in RCC4 in both normoxic and hypoxic conditions. However, the expression of CA IX in infected RCC4 cells was decreased (Figure 2A). This was also proven on the mRNA level by q-PCR (Figure 2B).

Since CA IX expression is regulated by HIF-1, we also looked at the expression of the α subunit of HIF and HIF targets, such as enolase and lactate dehydrogenase-A (LDH-A). As expected, HIF-1α was strongly expressed in the RCC4 cell line under both normoxic and hypoxic conditions. In infected cells, the expression of HIF-1α protein was lower than in the uninfected cells (Figure 2C). This was also proven by q-PCR on the transcription level (Figure 2D). In infected RCC4 cells, the level of other HIF targets such as LDH-A, an enzyme important in energy metabolism, and enolase, a metalloenzyme responsible for the catalysis of conversion of 2-phosphoglycerate to phosphoenolpyruvate in the glycolysis pathway, was also decreased (Figure 2E).

However, a preferred way of regulation in VHL-defective cells is by HIF-2α, which is active also under normoxic conditions [55]. Similarly, to HIF-1α, the expression of HIF-2α was high in uninfected cells independently of oxygen concentration and much lower in LCMV MX infected cells (Figure 2C).

From previous results, it is evident that the virus has a strong effect on the expression of CA IX. We wanted to find out if the viral NP, as the most abundant protein may cause these changes and whether the presence of only a single viral protein was enough to cause them.

We therefore transfected RCC4 cells with the viral NP protein alone. Western blot analysis confirmed the same effects on CA IX expression as after infection with a complete virus. The viral NP alone was sufficient to cause the changes in CA IX expression (Figure 2F).

### LCMV infection affects adhesion of RCC4 cells

E-cadherin and β-catenin are central molecules responsible for cellular adhesion. Loss or mutation of E-cadherin or signal transduction-related destabilization of E-cadherin-catenin interactions all result in reduced cell adhesion which is associated with metastasis and invasion [56, 57]. Therefore, we have analyzed the impact of LCMV infection on both, E-cadherin and β-catenin expression.

**Figure 3:**
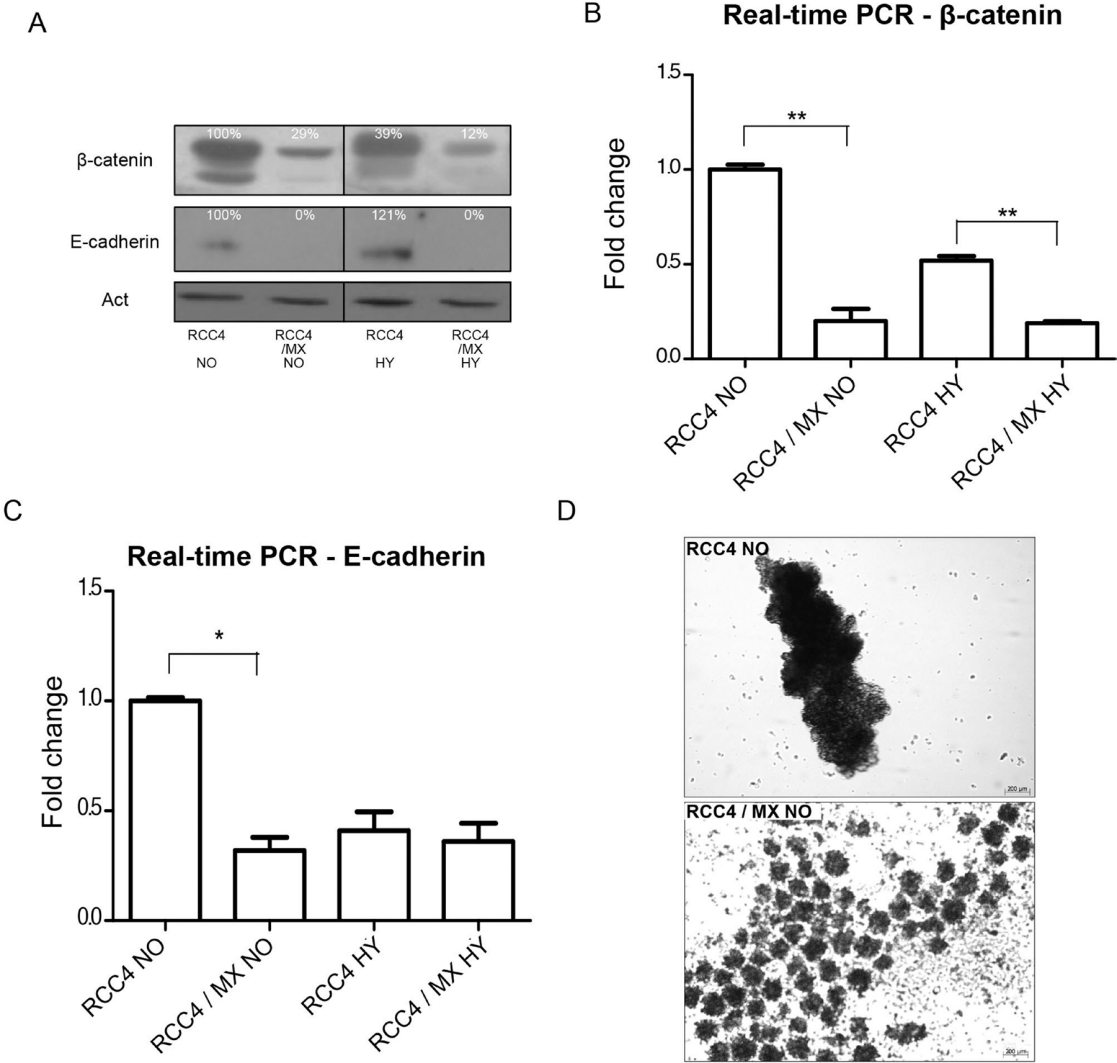
Analysis of adhesion molecules expression (**A**) Western blot analysis of β-catenin and E-cadherin in RCC4 and RCC4/MX cells under normoxic (NO) and hypoxic (HY) conditions. β-actin (Act) was used as a loading control. The numbers indicate percentage of densitometric evaluation. Q-PCR of (**B**) β-catenin and (**C**) E-cadherin in infected or uninfected cells cultivated under normoxic (NO) and hypoxic (HY) conditions. The samples were normalized to β-actin and ΔΔCt method was done to evaluate the fold change of infected and uninfected cells. Single and double asterisk indicates *p* value lower than 0.05 and 0.01, respectively, determined by the Student's *t*-test. (**D**) Aggregation assay of uninfected RCC4 and infected RCC4/MX cells. Cells (1.5 × 105 cells/well) were seeded into a plate with non-adhesive surface and incubated on a rotation shaker at 120 rpm for 24 h. The cell aggregates were observed by Zeiss Axiovert 40 CFL microscope, magnification 200×. Bar = 200 μm.

Western blot analysis of uninfected and infected RCC4 cells for the expression of E-cadherin and β-catenin showed that the expression of both proteins in infected RCC4 cells was lower than in non-infected control cells (Figure 3A). Also, the mRNA levels of these genes in infected cells were lower (Figure 3B, 3C). These findings are in line with aggregation assay we performed next. Only the cells with strong cell-cell contacts and adhesion molecules, cultivated for 24 h on a rotation shaker, form aggregates. The infected RCC4 cells with lower expression of both β-catenin and E-cadherin, formed only small and weak multicellular aggregates, while the non-infected cells with strong expression of both adhesion molecules formed single large aggregate (Figure 3D).

### LCMV infection changes the localization of CA IX in RCC4 cancer cells

The main determinant of successful immunotherapy is a sufficient amount of the immunotherapeutic target. The loss of the antigen from the membrane, by internalization or defects in expression, decreases the amount of the target. CA IX is used as one of the targets for treatment of RCC by immunotherapy. Therefore, we have also analyzed the location of CA IX in uninfected and infected cells. In these experiments, we used the VII/20 antibody, which internalizes similarly to the G250 antibody used in the on-going clinical trials.

**Figure 4:**
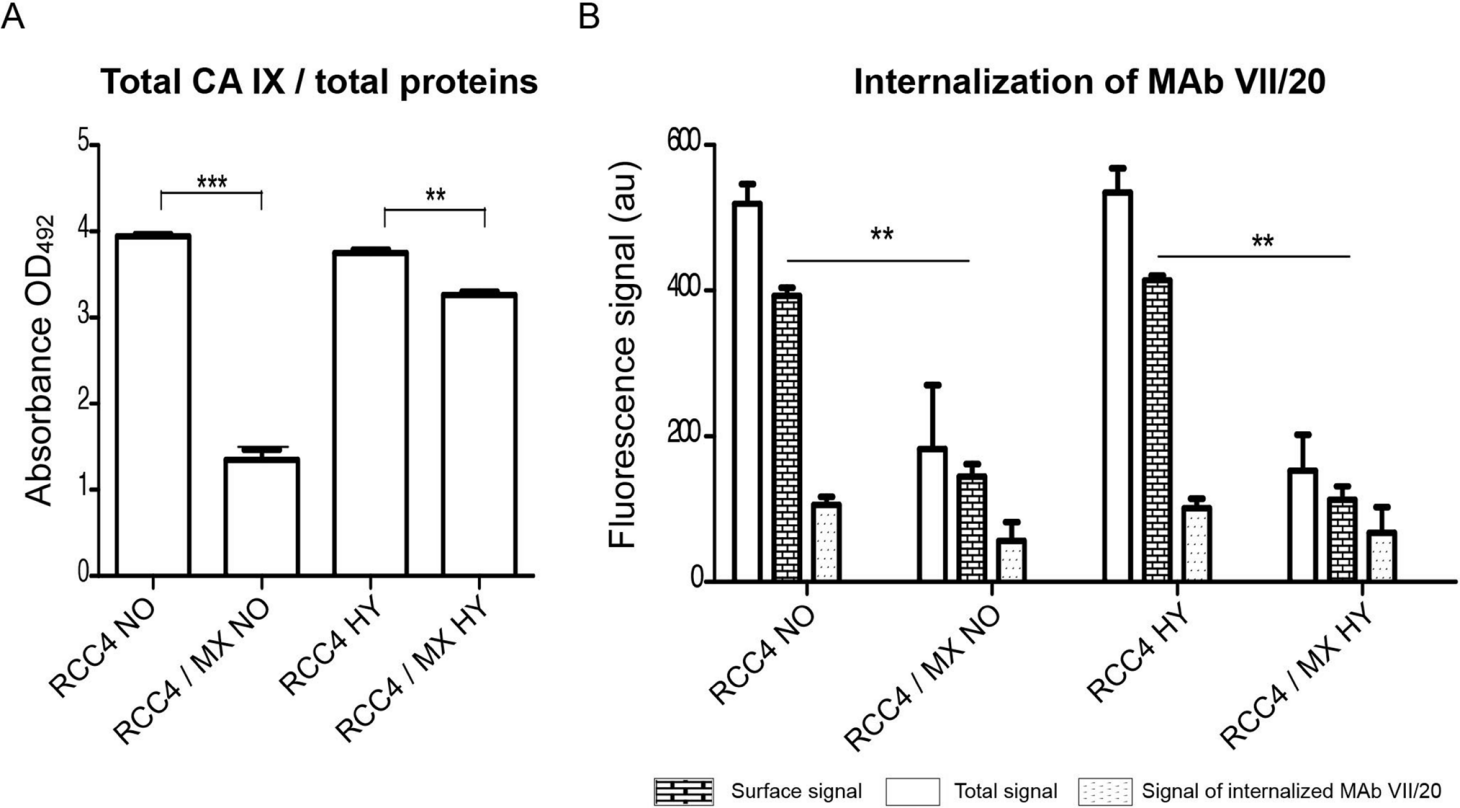
CA IX localisation and internalisation of VII/20 antibody in uninfected and infected cells (**A**) Determination of total level of CA IX present in uninfected and infected cells by ELISA. The total CA IX level was normalized to total amount of proteins. Y-axis shows the absorbance at OD_492_. Double and triple asterisk indicates *p* value less than 0.01 and 0.001, respectively, determined by the Student's *t*-test. (**B**) Flow cytometry of internalizing VII/20 antibody in uninfected and infected cells cultured in normoxia (NO) and hypoxia (HY). Y-axis shows fluorescence signal of VII/20 antibody. Empty bar shows total signal of the VII/20 antibody, bricked bar shows signal of only membrane CA IX bound VII/20 antibody, and dotted bar shows the signal of internalized VII/20 antibody. Double asterisk indicates *p*-value less than 0.01 determined by the Student's *t*-test.

The internalization of antibody can contribute to successful immunotherapy. We analyzed the distribution of CA IX in the cells by ELISA or flow cytometry by two different antibodies. Amount of total CA IX was determined by ELISA using the M75 antibody, while cell surface or internalized CA IX was determined by the VII/20 antibody in flow cytometry. As we showed earlier, the amount of total overall CA IX in the infected cells was significantly lower than in control uninfected cells (Figure 4A). There was also significantly less CA IX localized on the cell surface in the infected cells (Figure 4B). The amount of internalized VII/20 antibody was also lower in the infected cells (Figure 4B).

## DISCUSSION

Many commonly known viruses affect the tumor cells. The viruses might cause changes which may have a positive or negative impact on anti-cancer therapy. In this work, we identified a virus that might decrease the efficiency of immunotherapy.

A unique feature of LCMV strain MX is its reactivation from persistent infection during lower oxygen concentrations [20]. Persistence is typical for its asymptomatic course of infection without shedding of the infectious virus. In humans, hypoxic reactivation of the virus can occur during pregnancy, heart attack, stroke, ischemia, during organ transplantation or in tumors. After exposure to hypoxia, the virus reactivates and begins to shed infectious viral particles, and serious clinical symptoms might appear. This probably caused a series of death cases after organ transplantation [17–19] when undiagnosed persistent virus reactivated during partial hypoxia of the organ. In four cases of LCMV infection after transplantation all kidney recipients were diagnosed with LCMV present in the transplanted organ [17–19]. As renal cell carcinoma belongs to the most frequent cancers in the human population and simulates permanent hypoxic conditions due to VHL mutation, we were interested in the effect of LCMV strain MX infection on RCC4 cells.

Hypoxia inducible factor (HIF) acts as the main transcriptional regulator of over 100 genes that generally promote adaptation to low oxygen conditions (hypoxia). It is a heterodimeric transcription factor formed by association of an alpha subunit that is rapidly degraded in the presence of oxygen, and constitutively expressed beta subunit [58–60]. In hypoxic conditions, the alpha subunit is stabilized leading to its accumulation, and formation of the HIF complex. The complex recruits co-activators such as p300/CBP [61], and binds to hypoxia response elements (HRE) within the cis-acting regulatory elements of HIF target genes thereby directing their transcription. In VHL-defective tumors, like renal clear cell carcinoma, the HIF transcriptional program is constitutively activated.

Interestingly, VHL-defective RCC cells show an unusual inclination toward HIF-2α rather than HIF-1α expression [53, 55]. Many sources give evidence that HIF-2α, and not more intensively studied HIF-1α, acts as regulator in VHL-defective renal carcinomas [62]. Renal carcinoma cell lines and tumors produce both HIF-1α and HIF-2α or HIF-2α alone [53, 63]. Presence of HIF-2α in preneoplastic lesions correlates with upcoming malignancy [64]. However, HIF-2α, but not HIF-1α, can overcome VHL's tumor suppressor activity [65–67] and eliminating HIF-2α is sufficient to suppress tumor formation [68, 69]. Under certain conditions, despite their similarity, HIF-1α and HIF-2α can clearly antagonize one another. For example, in some models HIF-1α antagonizes, while HIF-2α potentiates c-Myc activity [63, 70]. Both proteins can also reciprocally regulate each other's protein levels where loss of HIF-1α leads to induction of HIF-2α and vice-versa [67]. Overproduction of HIF-1α in VHL-defective renal carcinoma cells suppresses tumor formation [67], while overproduction of HIF-2α promotes tumor growth [65, 67]. It was also reported that renal tumors with high HIF-1α but with low HIF-2α expression levels had worse overall survival compared to tumors expressing low levels of both, HIF-1α and HIF-2α [71]. Additionally It was proven that targeted knockdown of HIF-2α by siRNA enhanced HIF-1α protein levels [72]. In this work, we have shown that the expression of both, HIF-1α and HIF-2α in infected cells is lower.

We observed significant changes in the expression of CA IX after infection by LCMV strain MX, but further we were intrigued how the infection affected its pH-regulatory functions. CA IX plays an important role in regulation of extracellular and intracellular pH that is essential for survival of cancer cells. However, after the cultivation in normoxic or hypoxic conditions and measurement of extracellular pH, we found that the infection had no impact on pH regulation as the differences in pH between infected and uninfected cells were not significant (data not shown).

β-catenin connects E-cadherin with α-catenin and thereby to cytoskeleton and thus plays its role in the formation of adherent junctions between epithelial cells. The connection between E-cadherin and β-catenins is essential for cell-adhesive function. Destabilization of intercellular adhesion leads to deadhesion and migration of cells contributing thus to increased tumor aggressiveness and metastasis. In our RCC4 model, we have shown that the infected cells had lower levels of β-catenin and E-cadherin, which suggests that the infected cells might be more prone to metastasis. The same conclusions may be drawn from the aggregation assay.

In cancer immunotherapy, the distribution of antibody in the tumor is very heterogeneous, due to different factors like suboptimal targeting of some tumor regions, heterogeneity of expression of the tumor-associated antigen [73], tumor necrosis, vascular volume, blood flow rate, and vascular permeability [74]. Also a number of physiological barriers that contribute to poor localization of antibodies in tumors was identified: heterogeneous blood supply, centrally elevated interstitial fluid pressure, and large transport distances in the interstitium [75, 76]. All the mentioned factors vary in the tumor microenvironment from one location to another and from one day to the next [75], and also from patient to patient. Different therapeutic strategies may be required for each patient or sets of patients. In the presence of high levels of antigen, monotherapy with antibodies is sufficient, while in tumors with low expression of the target, combined therapy is needed.

Tumor uptake of the chimeric G250 monoclonal antibody directed against CA IX in patients with primary RCC is among the highest reported in solid tumors. The intratumoral distribution of the antibody is highly heterogeneous, therefore limiting the efficacy of the therapy [47]. However, the identical distribution of the two radiolabeled monoclonal antibody injections spaced 4 days apart indicates that the tumor parameters governing cG250 uptake do not significantly alter [47]. Also, antibody VII/20 was shown to overcome different drawbacks of the tumor microenvironment, such as high cellular density or low pH. The antibody was able to reduce the size of xenografts in mice, however, its distribution *in vivo* needs further analysis [52]. In this study we prove that the tumor-associated antigen CA IX is differentially expressed in cells infected with LCMV strain MX. The RCC4 cells infected with LCMV show lower overall and cell surface expression of CA IX, and therefore less target for the therapy with anti-CA IX antibodies. Infected cells also display a lower capability of CA IX internalization upon interaction with the antibody.

The presence of the virus leads to lower expression of both, HIF-2α and HIF-1α, and its targets such as LDH-A, enolase or CA IX. It also causes a decrease in overall and cell surface CA IX. Lower expression of CA IX, lower expression of adhesion molecules and aggravated aggregation of infected cells, could lead to decreased amount of target for immunotherapy and increased metastatic potential of the infected cells. This suggests that the LCMV-infected RCC4 cells might be more resistant to immunotherapy. The patients should therefore be tested for LCMV infection, and in the presence of virus, antiviral treatment should be applied before therapy with anti-CA IX antibodies.

In this work, we show that LCMV infection has a significant impact on renal carcinoma cells. This is one of many examples of why attention should also be paid to other factors, like infections, during cancer treatment. Knowledge about the influence of tumor-associated infections may lead to successful treatment of cancer.

## MATERIALS AND METHODS

### Virus and cell lines

The renal carcinoma cell line RCC4 is derived from human renal carcinoma lacking functional protein VHL showing constitutive elevation of HIF-1α protein under normoxic conditions. RCC4 VHL cell line stably transfected with pcDNA3-VHL was obtained from Dr. Patrick Maxwell [53]. The cells were cultivated in Very Low Endotoxin Dulbecco's MEM medium containing stable 2 mM L-glutamine (Biochrom, Berlin, Germany) with 10% fetal calf serum (FCS, Biochrom) and gentamicin 80 μg/ml (Lek, Ljubljana, Slovenia) at humidified atmosphere at 37°C in the presence of 5% CO_2_. All the cultivations under hypoxic conditions were done in hypoxic workstation (Ruskin Technology, Bridgend, United Kingdom) in a mixture of gases with 2% O_2_ 5% CO_2_, 2% H_2_, and 91% N_2_ at 37°C for 24 h. RCC4 and RCC4 VHL cell lines were infected by persistent LCMV strain MX using cell-free extracts and designated as RCC4/MX and RCC4 VHL/MX.

### Infection by cell-free extracts

Infection by cell-free extracts was done according to the procedure of Laposova [54]. Briefly, one ∅ 10 cm tissue culture dishes with monolayer of persistently infected BHK/MX were washed once by cold PBS (Sigma Aldrich) and scraped into 400 μl of deionized H2O and incubated for 15 min on ice with repeated shaking. The cells were then centrifuged at 900 rpm at 4°C for 15 min. Supernatant was diluted in 1:1 ratio with DMEM containing 4% FCS, filtered through 0.2 μm filter and transferred onto RCC4 cells. The following day the extract was exchanged for DMEM with 10% FCS. The infected cells were left to grow to high density to allow for easier virus transmission via cell-to-cell contacts.

### Proliferation assay

One thousand RCC4 cells and infected RCC4/MX cells were plated to 24 well plates in triplicates and the cell concentration was measured every 12 h by cell counter Beckman coulter Z2 (Beckman Coulter Life Sciences, Indianapolis, USA).

### Isolation of total RNA and reverse transcription

Cell monolayers grown under normoxic or hypoxic conditions for 24 h were washed with ice-cold PBS and total RNA was extracted with InstaPure reagent (Eurogentec, Seraing, Belgium) according to manufacturer's protocol. The cells were scraped and transferred into new eppendorf tube. Two hundred and fifty μl of chloroform (Slavus, Bratislava, Slovakia) were added and incubated on ice for 15 min, followed by centrifugation at 14,500 rpm for 15 min. Supernatant was transferred to a new eppendorf tube and isopropanol (Slavus) in ratio of 1:1 was added. After 20 min incubation at –80°C, the samples were centrifuged at 14,500 rpm. The pellets were washed by centrifugation at 7,500 rpm with 70% and 96% ethanol, respectively and air dried for 10 min. The pellets were dissolved in 10 μl of DEPC water and RNA concentration was measured on NanoDrop 2000 (Thermo Scientific, Wilmington, USA).

For reverse transcription we used 2000 ng of total RNA mixed with 10 μl of master mix (High capacity cDNA reverse transcriptase kit, Applied Biosystems, Carlsbad, USA). Master mix contained 5× RT buffer, 100 mM random primers, 100 mM dNTP and DEPC water. Conditions for the reaction were as follows: 25°C for 10 min, 37°C for 120 min and 85°C for 5 min.

### PCR

To detect different viral genes, we used different sets of primers (Microsynth AG, Balgach, Switzerland). Primers for viral NP, forward: AAA TAC CCA AAT CTC AAT GAC CTT GA and reverse: CCT ACA AGC TAT GTA TGG CCA CC; for GP, forward: AAC CAG TGC AGA ACT TTT AGA GGT A and reverse: GCA AGT CTT CTA GTG AGG AAC TTT G; for ZP, forward: CCT GTG AGA GTA CAG AGA and reverse: GAT ATC TTC AGC TTG GTT; for L protein, forward: AGC TGC TGT CTC GTT GTA TAG AAA T and reverse: ATA CAT GCC AAC TTG TTA GTG TCC T. We used Dream Taq Green PCR master mix (Thermo Scientific, Foster City, USA) with 100 mM specific primers, 1 μl of cDNA and water. The conditions for PCR were the same for all the genes: 95°C 3 min, 95°C 30 sec, 60°C 40 sec, 72°C 40 sec repeated 35 times and a final polymerization at 72°C for 7 min.

### Quantitative PCR

Quantitative PCR was performed on a StepOne real-time PCR system (Applied Biosystems, Foster City, CA.) using Maxima SYBR green/ROX q-PCR master mix (Thermo Scientific, Eugene, USA) with 3 μl of 10× diluted cDNA, 100 mM specific primers for carbonic anhydrase 9 : forward: TAT CTG CAC TCC TGC CCT CTG, and reverse: CAC AGG GTG TCA GAG AGG GTG T; for HIF-1α : forward: CAA GTT GGA ATT GGT AGA AAA ACT T, and reverse: CGG TCT TTT GTC ACT GTT TTT AAT T; for E- cadherin: forward: CAC AGA TGG TGT GAT TAC AGT CAA, and reverse: CCC AGT CTC TCT TCT GTC TTC TGA; for β-catenin: forward: GCT GAT TTG ATG GAG TTG GAC ATG G, and reverse: GCC AAA CGC TGG ACA TTA GTG G; and for actin forward: CCA ACC GCG AGA AGA TGA CC, and reverse: AGG ATC TTC ATG AGG TAG TCA GTC genes. Thermal profile for q-PCR was the same as for conventional PCR. All samples were analyzed in triplicates. Sample Ct values were normalized to β-actin as internal control. Relative expression was calculated using ΔΔCt method. Results were analyzed with two-tailed unpaired *t* test (Student test) with a *P*-value of < 0.05 considered significant.

### Transient transfection

The cells were plated onto ∅ 30 mm tissue culture dish to reach density of approximately 70% on the following day. Transfection was performed with 4 μg of plasmid DNA (pcDNA 3.1.-NP and pcDNA3.1 empty vector) diluted in 400 μl of serum free medium and 6 μl of Turbofect transfection reagent (Thermo Scientific, Vilnius, Lithuania) and incubated for 20 min at room temperature. The reaction mixture was added drop-wise to the cells and the cells were incubated for 24 h at 37°C. The medium (3 ml DMEM with 10% FCS) was changed after 24 h and the following day, the cells were used for protein extraction. Protein extraction was done with 50 μl of ice-cold RIPA buffer (Sigma-Aldrich) supplemented with inhibitors of proteases (Roche Diagnostics GmbH, Mannheim, Germany) per culture dish and incubated on ice for 15 min. The cells were scraped into the eppendorf tube and centrifuged at 14,500 rpm for 15 min. Supernatant was transferred into new eppendorf tube and used for western blot analysis.

### Antibodies

Primary antibodies: We have used undiluted hybridoma medium of mouse monoclonal antibody M87, specific for nucleoprotein of LCMV strain MX [15, 77], undiluted hybridoma medium of mouse monoclonal antibody M75 (specific for the PG domain of the CA IX protein) [77] and VII/20 antibody (internalizing monoclonal antibody, specific for CA domain of the CA IX protein) [51]. Purified mouse anti-human HIF-1*α* antibody diluted 1:500 (BD Transduction, San Jose, CA, USA); rabbit anti HIF-2*α* antibody diluted 1:500 (Novus Biologicals, Littleton, CO, USA); mouse anti-enolase antibody in concentration 2 μg/ml (Abcam, Cambridge, UK); rabbit anti-lactate dehydrogenase-A (LDH-A) antibody diluted 1:1000 (Abcam), rabbit anti-*β*-catenin antibody diluted 1:500 (Santa Cruz Biotechnology, Santa Cruz, CA, USA), rabbit anti-E-cadherin antibody diluted 1:500 (Santa Cruz Biotechnology) and mouse anti β-actin diluted 1:5000 (Cell Signalling Technology, Danvers, MA, USA) were also used.

Secondary antibodies: polyclonal goat anti-mouse immunoglobulins conjugated with horseradish peroxidase, diluted 1:5000 (Dako, Glostrup, Denmark) and polyclonal swine anti-rabbit immunoglobulins conjugated with horseradish peroxidase, diluted 1:5000 (Dako). Donkey anti-mouse IgG (H + L) secondary antibody conjugated with Alexa Fluor 488 (Termo Fisher Scientific, Paisley, Scotland).

### Immunofluorescence

Cells were grown on glass coverslips, washed with PBS and fixed with ice-cold methanol at −20°C for 5 min. Nonspecific binding was blocked by incubation with PBS containing 3% bovine serum albumin (BSA, Applichem, Darmstadt, Denmark) for 1 h at room temperature. Fixed cells were incubated with primary antibody for 1 h at 37°C, and subsequently, the cells were washed 3 times for 10 min with PBS containing 0.2% Tween 20 (Sigma-Aldrich, Saint Louis, USA) and followed by incubation with secondary antibody diluted 1:1000 in PBS containing 1% BSA for 1h at 37°C. Samples were washed 3 times for 10 min in dark. The DAPI (4′, 6′-diamidino-2-phenylindole; Sigma-Aldrich, Saint Louis, USA) staining for nuclei was done at RT for 5 min. Cells were washed, mounted on slide and analyzed under confocal microscope. Zeiss LSM510 laser scanning confocal microscopy system mounted on a Zeiss Axiovert 200 M inverted microscope (Zeiss, Jena, Germany) was used. Images were taken with Plan Apochromat 63×/1.4 oil objective and scanned at scan speed 6, 1024 × 1024 pixels, 12 bit data depth with average mode 8× line.

### Western blot

One million of cells from each cell line were plated in duplicates on the ∅ 6 cm tissue culture dishes and incubated for 24 h under normoxic and hypoxic conditions. After 24 h protein lysates were prepared by scraping the cells into 100 μl of ice-cold RIPA buffer supplemented with inhibitors of proteases. The cells were incubated on ice for 15 min and centrifuged at 14,000 rpm at 4°C for 15 min. The concentrations of proteins were quantified by Pierce BCA protein assay kit (Thermo Scientific, Rockford, USA) and 100 μg of total proteins with 2× Laemeli buffer and 10% beta-mercaptoethanol were loaded onto 10% SDS-PAGE and run over night at 40V.

Proteins were transferred onto the PVDF membrane (Immobilon; Millipore, Billerica, USA) for 3 h at 300 mA. Membranes were blocked for 1 h in blocking buffer (5% nonfat dry milk in PBS containing 0.2% Nonidet P40 substitute Bioxtra, mixture of 15 homologues (Sigma Aldrich)) on the shaker for 1 h at RT, then the membranes were washed with washing buffer (PBS containing 0.2% Nonidet P-40). The membrane was incubated with primary antibody for 1 h at RT, washed 3 times for 15 min with washing buffer and followed by incubation with appropriate secondary antibody conjugated with horseradish peroxidase for 1 h at RT. The signal was developed with the ECL detection system (Pierce, Rockford, IL, USA). Densitometric analysis of western blots was done in ImageJ software [78], normalized to β-actin and expressed in percents.

### Aggregation assay

Cells (150,000 cells/well) were plated on a 24-well plate with a non-adhesive surface. The plates were shaken for 24 h on a rotary shaker at 120 rpm at 37°C and 5% CO_2_. The following day, the cells were analyzed on Leica DM4500B upright fluorescent microscope with camera Leica DFC480 (Leica, Wetzlar, Germany).

### Internalization

Cells, in concentration of 6 × 10^3^ cells were plated on to 6-well plate in triplicates and incubated for 24 h under normoxic and hypoxic conditions. Medium was replaced with medium containing internalization antibody VII/20. The incubation was done for 30 min either at 4°C where internalization process of the antibody is inhibited or at 37°C were the internalization occurs. The control cells were incubated with medium containing 10% FCS only. After the incubation, cells were used for flow cytometry.

### Flow cytometry

For flow cytometry the cells were washed 2 times for 10 min with PBS pH 7.2 and to all cells 1 ml of versene solution (Lonza, Verviers, Belgium) per well was added and incubated for 5 min at 4°C. The cells were scraped and transferred in to cooled eppendorf tubes and centrifuged at 2000 rpm for 7 min at 4°C. Supernatant was removed and pellet was resuspended in 100 μl of versene solution followed by drop-wise addition of 900 μl of 70% ethanol. The cells in ethanol were incubated on the rotation shaker for 1 h at 4°C, then washed twice by versene solution and centrifuged for 10 min. Centrifugation was followed by another incubation on ice for 10 min. The cells were labeled with 200 μl of secondary antibody conjugated with alexa flour 488 in dilution 1:1000 in DMEM with 10% FCS for 30 min at 4°C in the dark. Finally, the cells were washed twice by versene solution and centrifuged. Samples were analyzed by Guava Easy Cyte plus flow cytometer (Guava technologies-Merck, Darmstadt, Germany). Results were analyzed with two-tailed unpaired *t* test (Student test) with a *P*-value of < 0.05 considered significant.

### ELISA

Fifty thousand cells were seeded in six parallels onto 96-well plate and incubated under normoxic or hypoxic conditions for 24 h.

Analysis of surface CA IX protein on live cells: The cells were incubated with M75 antibody [77] (10 μg/μl in DMEM with 10% FCS, 50 μl/well) at 37°C for 2 h. The cells were quickly washed twice with PBS pH 7.2 and then incubated with secondary antibody (1:5000; 50 μl/well). After the incubation the samples were again washed 3× with PBS pH 7.2. The bound antibody was visualized by 10 mg of o-phenylenediamine substrate (Sigma-Aldrich) with 10 μl H_2_O_2_ in citric buffer (pH 5) for 5–15 min in the dark. The reaction was stopped by 10 mM H_2_SO_4_ (50 μl/well) and absorbance was measured at 492 nm in Synergy HT reader (Bio Tek instruments, Winooski, VT, USA).

Analysis of total CA IX on fixed cells: The cells were washed twice with PBS pH 7.2 and then fixed with methanol for 5 min at –20°C and washed twice again. Non-specific binding was blocked with 10 % FCS in DMEM medium at 37°C for 30 min (50 μl/well). Following steps were identical with the protocol on live cells.

## Abbreviations

CA IX: carbonic anhydrase IX
ENO: enolase
HIF-1α: hypoxia inducible factor-1α
LCMV: lymphocytic choriomeningitis virus
LDH-A: lactate dehydrogenase-A
NP: nucleoprotein
RCC: renal cell carcinoma
VHL: von Hippel-Lindau tumor suppressor gene
